# Acute pneumonitis consequent on pleurodesis with *Viscum album* extract: severe chest images but benign clinical course

**DOI:** 10.1186/2049-6958-9-61

**Published:** 2014-11-27

**Authors:** Suk Ju Cho, Su Wan Kim, Jee Won Chang

**Affiliations:** Department of Anesthesiology and Pain Medicine, Jeju National University Hospital, Jeju National University School of Medicine, Jeju, Korea; Department of Thoracic and Cardiovascular Surgery, Jeju National University Hospital, Jeju National University School of Medicine, Aran 13 gil 15, Jeju-si, Jeju Special Self-Governing Province 690-767 Korea

**Keywords:** Computed tomography, Lung infection, Pneumothorax, Segmentectomy

## Abstract

Chemical pleurodesis is widely recommended in the treatment of pulmonary air leak of different etiologies as well as malignant pleural effusions and chylothorax. Conventional chemical pleurodesis using erythromycin, tetracycline, hydrophilic fumed silica, autologous blood and talc slurry has been standardized, and its complications, including high fever, intractable chest pain, and acute lung injury, seem to be frequent. *Viscum album* extract is a new chemical agent for pleurodesis, and only a few studies have reported outcomes of such chemical pleurodesis in the treatment of malignant pleural effusion. Moreover, the complications resulting from pleurodesis using *Viscum album* extract are very rare, and acute pneumonitis has not been reported. in this paper we report the first case of acute pneumonitis after pleurodesis using *Viscum album* extract in a 58-year-old man who had prolonged air leaks after a left upper lingularsegmentectomy for metastatic lung cancer. We performed repeated pleurodesis four times with 2 to 4 days intervals. While the patient had no symptoms of pneumonia, such as cough, sputum, chilling, and fatigue, a follow-up chest X-ray revealed increasing peribronchial consolidations and infiltrations in the left upper lobe. A chest tomography showed extensive parenchymal consolidations and ground-glass appearances in the left lungs, representing pneumonia with acute lung injury. The acute pneumonitis was spontaneously resolved with supportive care, and the patient was discharged ten days after the development of pneumonitis. We think that pleurodesis with *Viscum album* extract is effective, but repeated pleurodesis should be avoided for possible onset of acute pneumonitis.

## Background

Acute pneumonitis is a frequent problem that has a great impact on the quality of life of patients treated with pleurodesis. It has been suggested that acute pneumonitis has been caused by pulmonary deposition and trans-pleural absorption of the sclerosing agents, such as tetracyclin, bleomycin, and talc, and it occurs through lymphatic stomata, whose openings are distributed in the parietal pleural [1]. However, the pathophysiologic mechanism responsible for this severe complication is still unclear. To date, most therapeutic options have been limited to supportive care. *Viscum album* extract from European mistletoe contains a number of biologically active compounds, mainly the mistletoe lectins, viscotoxins, and other low molecular weight proteins, which exert immune-modulatory, cytotoxic, apoptotic, and anti-angiogenic effects [2]. This extract is the most frequently prescribed drug for complementary treatment in cancer patients in several European countries. However, only a few studies have reported outcomes after its use for chemical pleurodesis in the treatment of malignant pleural effusion [3–5] and any serious side effect, such as acute pneumonitis, has not been described yet. We hereby report the first case of acute pneumonitis, an unusual complication after pleurodesis with *Viscum album* extract.

## Case presentation

**Figure 1:**
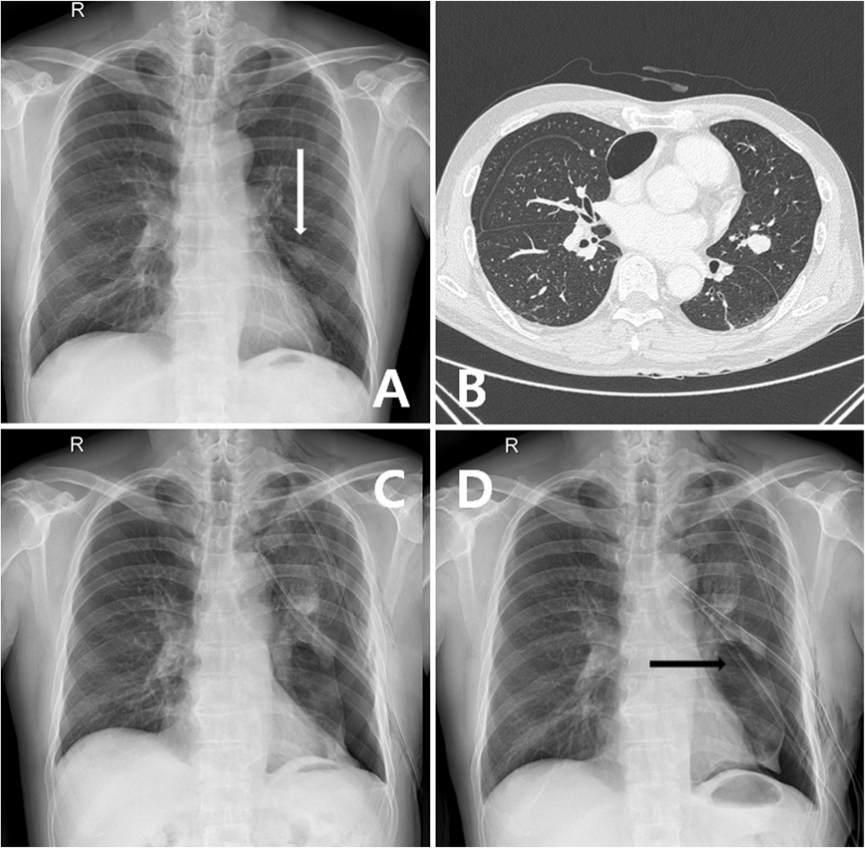
**Peri-operative X-ray findings.** Chest X-ray **(A)** and computed tomography **(B)** show a metastatic lung cancer. A post-operative persistent air leakage results in collapse of the left lungs **(C)**. While we added a chest tube, the lungs could not be fully expanded **(D)**.

A 58-year-old male patient, who had undergone a left nephrectomy for renal cell carcinoma five years before, was admitted to our department because a routine chest CT showed a pulmonary solitary nodule. Chest X-ray and CT revealed a well-circumscribed mass in the left middle lung field that was suspicious for metastatic lung cancer (Figure 1A,B). We performed a lingular segmentectomy through a thoracotomy. The patient had an incomplete fissure between the upper and lower lobes, and we used two auto-stapling devices to separate the fissure. We inserted two chest tube drainages and then closed the thoracotomy. The post-operative course was complicated by persistent air leakage resulting in collapse of the left lung, as shown in Figure 1C. While we added a chest tube four days after the operation and kept drainage with negative pressure, the lungs could not be fully expanded (Figure 1D).

**Figure 2:**
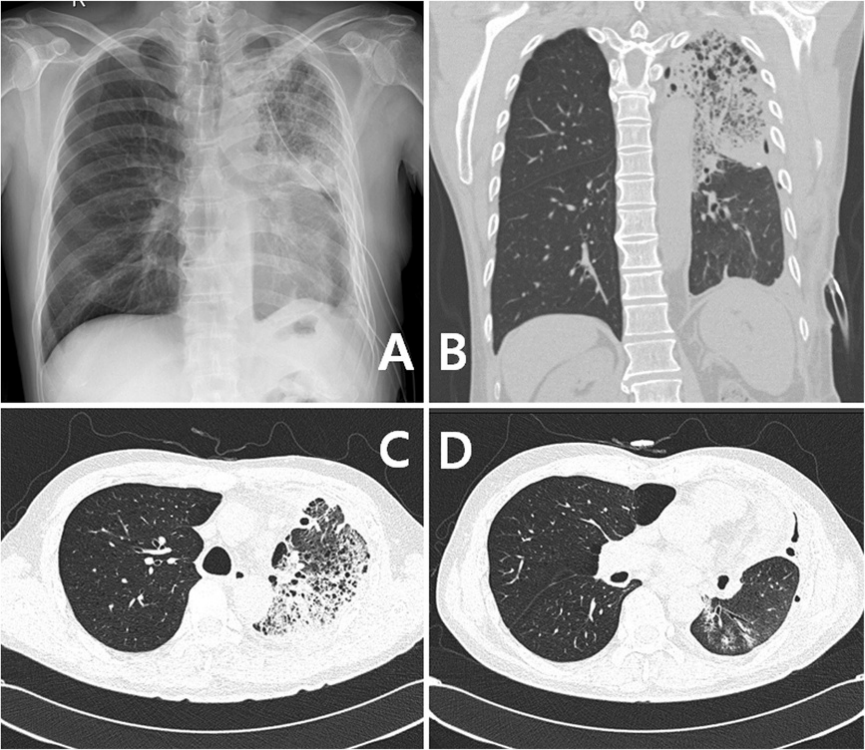
**Images of acute pneumonitis.** A follow-up chest X-ray shows increasing extents of peribronchial consolidations and infiltrations in the left upper lung **(A)**. A chest computed tomography reveals extensive parenchymal consolidations in the whole left lungs, air-fluid levels within the emphysematous bullae, and multiple reactive mediastinal lymph nodes; all of these findings indicated a suspicious of pneumonia **(B, C, D)**.

We underwent a chemical pleurodesis using an extract of *Viscum album* (European mistletoe) (ABNOBA viscum F®, ABNOBA Heilmittel GmbH, Pforzheim, Germany) six days after the operation. We mixed five ampoules of ABNOBA viscum F® (100 mg) and 200 ml normal saline, and injected them through the chest tube. We repeated the pleurodesis eight days and twelve days after the operation with the same method. Then we removed one chest tube because of decreasing amount of air leakage. The fourth pleurodesis was performed sixteen days after the operation. Unfortunately, a follow- up chest X-ray showed increasing extents of peribronchial consolidations and infiltrations in the left upper lung (Figure 2A). A chest CT revealed extensive parenchymal consolidations in the whole left lung, air-fluid levels within the emphysematous bullae, and multiple reactive mediastinal lymph nodes; all of these findings indicated a suspicious of pneumonia (Figure 2B,C, and D).

**Figure 3:**
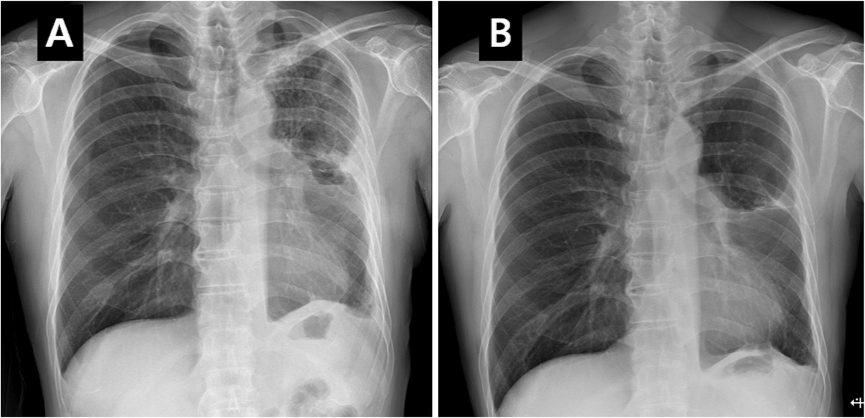
**X-ray findings of resolving the acute pneumonitis.** A follow-up chest X-ray showed a markedly decreased pulmonary infiltrations so as the remaining chest tube was removed **(A)**. The patient was discharged twenty-six days after the segmentectomy and followed- up for four months without any respiratory symptoms **(B)**.

On the other hand, the patient had no subjective symptoms, such as febrile sense, dyspnea, cough, and sputum. An arterial blood gas analysis revealed a pH of 7.39, a PaCO_2_ of 39.7 mmHg, a PaO_2_ of 87 mmHg, and oxygen saturation of 93.1% on nasal prong oxygen 2 L/min. A white blood cell count was 17,200 /*u*L with 78.6 % of segmented neutrophil. C-reactive protein was 30.5 mg/dl, and procalcitonin level was 2.94 mg/dL. Blood chemistry showed serum creatinine of 1.6 mg/dL, potassium of 5.7 mmol/L, serum albumin of 2.9 mg/dL, and alanine transaminase of 16 IU/L. We performed a supportive care including oxygen inhalation, mucolytics, diuretics, and empirical antibiotics (moxifloxacin). Five days after the supportive care, a follow- up chest X-ray showed markedly decreased pulmonary infiltrations, and the remaining chest tube was removed (Figure 3A). The patient was fully recovered from the acute pneumonitis with normalized laboratory findings and oxygen saturation of 98% at room air. The patient was discharged twenty-six days after the segmentectomy and followed-up for four months without any respiratory symptoms (Figure 3B).

## Discussion

Prolonged air leak is one of the most common post-operative complications encountered after thoracic surgery that involves that involves mobilization or pulmonary resection. It is an important complication that results in an increased hospital stay for prolonged chest tube drainage and is associated with other post-operative complications, such as thoracic pain, pneumonia and respiratory distress [6, 7]. Rena et al. suggested that a routine use of surgical staplers, even buttressed by synthetic or organic strips, does not result in an adequate air sealing in most patients undergoing pulmonary lobectomy [8]. When an air leak persists after pulmonary lobectomy, a bronchoscopy is generally recommended to rule out broncho-pleural fistula. A significant broncho-pleural fistula should be corrected by surgical intervention, including re-closure of bronchial stump and reinforcement with surrounding pericardium, muscle, or soft tissue. We performed bronchoscopy two days after segmentectomy for prolonged air leakage. There was no evidence of broncho-pleural fistula. However, lots of blood-tingled secretions in the bronchial stump and inflammatory changes were observed. After removing the secretions, the left lower lobe appeared to be slightly re-expanded. The left lung was re-collapsed the next day. Although we added a chest tube four days after the operation and kept drainage with negative pressure, the left lung could not be fully expanded. Therefore, we decided to perform chemical pleurodesis.

Chemical pleurodesis is widely recommended in the treatment of pulmonary air leak of different etiologies as well as malignant pleural effusions and chylothorax [7, 9–11]. Instillation of a sclerosing agent into the pleural space elicits an inflammatory reaction in the pleura that allows for the obliteration of the pleural space and resolution of an air leak or pleural effusion. Numerous sclerotic agents, including erythromycin, tetracycline, hydrophilic fumed silica, autologous blood and talc slurry [9, 12–14] have been used to produce pleural symphysis, and several studies have investigated the efficacy and complications of them. Reported usual minor complications of pleurodesis are high fever, severe pain, tachycardia, and dyspnea. While talc is the most commonly used sclerosing agent and known to be the most effective material, there have been variable reports of its serious complications, such as pulmonary edema, acute pneumonitis, and acute respiratory distress syndrome [15–18]. For these reasons, several investigations to search for new agents for pleurodesis are ongoing. Byun et al. researched effects of taurolidine and proposed that it is an effective alternative agent for the management of pulmonary air leakage [19]. Godazandeh et al. and other researchers suggested that povidone-iodine is an effective, inexpensive, safe and feasible agent for chemical pleurodesis in the management of malignant pleural effusion [20]. These new agents seem to need further investigation because there was a report about serious complication of visual loss after pleurodesis using povidone-iodine [21].

ABNOBA viscum F® is an extract of *Viscum album* (European mistletoe) which grows on trees of the genus Fraxinus. This extract is the most frequently prescribed drug for complementary treatment in cancer patients in several European countries. It is known to stimulate the immune system, improve survival, enhanc equality of life, and decrease the side effects of chemotherapy and radiotherapy [22, 23]. However, only few studies have reported outcomes after its use for chemical pleurodesis in the treatment of malignant pleural effusion [3–5] and congenital chylothorax [24]. Furthermore, these studies do not report any serious complications, only slight side effects such as mild febrile sense, scanty burning, and loculated effusion.

In our case, while the images of the acute pneumonitis revealed grave findings that resembled acute respiratory distress syndrome, the patient presented no subjective symptom. The supportive cares, including oxygen inhalation, mucolytics, diuretics, and empirical antibiotics, could fully recover the patient ten days after the acute pneumonitis. We repeated pleurodesis four times with 2 or 4 days intervals and mixed five ampoules of ABNOBA viscum F® (100 mg) and 200 ml normal saline, and we injected them through the chest tube. The dosage of *Viscum album* extract was selected based on the dosage for adults with a body weight of 60 Kg. The usual starting dose is 100 mg and it can be increased to 500 mg [3, 5]. Because the dosage has a wide range, we suggest that the dosage should not result in an acute pneumonitis. We suppose that the interval of repeated pleurodesis is potentially related to the acute pneumonitis.

## Conclusions

A pleurodesis with *Viscum album* extract, for patients with malignant pleural effusion and prolonged air leakage after lung resection and chylothoraxm, seems to be effective. We propose that, when repeated pleurodesis is mandatory, pleurodesis with *Viscum album* extract should be performed with careful attention and at sufficient intervals over one week. If an acute pneumonitis occurs, it can be spontaneously and quickly resolved with supportive care. However, there are not enough data in literature to prove the efficacy or to represent complications of the pleurodesis using *Viscum album* extract. We believe that a treatment strategy for pleurodesis using *Viscum album* extract can be feasible after further variable and intensified studies.

## Consent

Written informed consent was obtained from the patient for publication of this case report and any accompanying images. A copy of the written consent is available for review by the Editor-in-Chief of this journal.
